# Posttraumatic Stress Symptom Trajectories in Family Caregivers of Patients With Acute Cardiorespiratory Failure

**DOI:** 10.1001/jamanetworkopen.2023.7448

**Published:** 2023-04-07

**Authors:** Blair Wendlandt, Liam Pongracz, Feng-Chang Lin, Mark Toles, Bradley N. Gaynes, Laura C. Hanson, Shannon S. Carson

**Affiliations:** 1 Division of Pulmonary Diseases and Critical Care Medicine, Department of Medicine, University of North Carolina at Chapel Hill; 2 Department of Biostatistics, University of North Carolina, Chapel Hill; 3 School of Nursing, University of North Carolina at Chapel Hill; 4 Department of Psychiatry, UNC School of Medicine, University of North Carolina at Chapel Hill; 5 Department of Epidemiology, Gillings School of Global Public Health, University of North Carolina at Chapel Hill; 6 Division of Geriatric Medicine and Palliative Care Program, Department of Medicine, University of North Carolina at Chapel Hill

## Abstract

**Importance:**

Overall, 1 in 3 family caregivers of patients who require intensive care unit (ICU) admission will experience significant posttraumatic stress symptoms (PTSSs), but little is known about how PTSSs evolve over time. Measuring PTSS trajectories could facilitate the development of targeted interventions to improve mental health outcomes for family caregivers of critically ill patients.

**Objective:**

To measure 6-month PTSS trajectories among caregivers of patients with acute cardiorespiratory failure.

**Design, Setting, and Participants:**

This prospective cohort study was conducted in the medical ICU of a large academic medical center among adult patients requiring (1) vasopressors for shock, (2) high-flow nasal cannula, (3) noninvasive positive pressure ventilation, or (4) invasive mechanical ventilation. Each patient was enrolled along with their primary caregiver, ie, the unpaid individual who provided the most physical, emotional, or financial support prior to ICU admission.

**Main Outcomes and Measures:**

Family caregiver PTSSs were assessed using the Impact of Events Scale–Revised within 48 hours of ICU admission, following ICU discharge, and 3 and 6 months after enrollment. Latent class growth analysis was used to measure PTSS trajectories. Preselected patient and caregiver characteristics measured at ICU admission were analyzed for association with trajectory membership. Six-month patient and caregiver outcomes were analyzed by caregiver trajectory.

**Results:**

Overall, 95 family caregivers were enrolled and provided baseline data; mean (SD) age was 54.2 (13.6) years, 72 (76%) were women, 22 (23%) were Black individuals, and 70 (74%) were White individuals. Three trajectories were identified: persistently low (51 caregivers [54%]), resolving (29 [31%]), and chronic (15 [16%]). Low caregiver resilience, prior caregiver trauma, high patient severity of illness, and good patient premorbid functional status were associated with the chronic trajectory. Caregivers with the chronic PTSS trajectory had worse 6-month health-related quality of life (mean [SD] total 36-item Short Form Survey score, persistently low trajectory: 104.7 [11.3]; resolving trajectory: 101.7 [10.4]; chronic trajectory: 84.0 [14.4]; *P* < .001) and reduced effectiveness at work (mean [SD] perceived effectiveness at work score, persistently low trajectory: 86.0 [24.2]; resolving trajectory: 59.1 [32.7]; chronic trajectory: 72.3 [18.4]; *P* = .009).

**Conclusions and Relevance:**

In this study, 3 distinct PTSS trajectories among ICU family caregivers were observed, with 16% of caregivers experiencing chronic PTSSs over the subsequent 6 months. Family caregivers with persistent PTSS had lower resilience, prior trauma, higher patient severity of illness, and higher baseline patient functional status compared with family caregivers with persistently low PTSS, with adverse effects on quality of life and work. Identifying these caregivers is an essential first step to develop interventions tailored to those with the greatest need for support.

## Key Points

**Question:**

How do posttraumatic stress symptoms (PTSSs) experienced by family caregivers of patients with acute cardiorespiratory failure evolve over the 6 months following intensive care unit admission?

**Findings:**

In this cohort study of 95 family caregivers, latent class analysis found that 16% of caregivers experienced chronic PTSSs, which was associated with reduced quality of life and diminished effectiveness at work. Higher patient severity of illness, better patient functional status, higher caregiver resilience, and prior caregiver trauma were associated with the chronic PTSS trajectory.

**Meaning:**

These findings suggest that caregivers who are at risk for chronic PTSSs can be identified early and may benefit from interventions targeting low resilience and history of trauma.

## Teaser

This cohort study measures 6-month posttraumatic stress symptom trajectories among caregivers of patients with acute cardiorespiratory failure treated in the intensive care unit.

## Introduction

Critical illness has significant health consequences for patients and their families. Posttraumatic stress symptoms (PTSSs) are common among family caregivers of patients who have experienced critical illness, and approximately 1 in 3 caregivers will be affected.^1,2^ Potential contributors to PTSSs for caregivers of patients in the intensive care unit (ICU) include witnessing near or actual patient death, fear and worry about the patient’s health, distrust of medical professionals, and poor patient health following ICU discharge.^2,3,4,5,6^ PTSSs are associated with negative outcomes in the general population, including marital difficulties, occupational challenges, substance abuse, suicide attempts, and mortality.^7,8^ Furthermore, evidence suggests that caregiver PTSSs may be associated with adverse health outcomes for both caregiver and patient.^9^ A working group for the National Heart, Lung, and Blood Institute has identified interventions to reduce PTSSs and related forms of psychological distress for caregivers as a research priority to improve long-term outcomes for both ICU caregivers and patients.^10^

Several clinical trials of communication and support interventions have been unsuccessful in reducing PTSSs among ICU caregivers.^11,12,13^ One possible explanation is that caregivers were deemed eligible for intervention based only on high patient severity of illness, without considering individual caregiver risk for posttraumatic stress. Research among individuals who have experienced other traumatic events, such as serious injury or military combat, has shown that risk varies widely for onset, duration, and trajectory of PTSSs. Some individuals will never develop PTSSs, some will develop PTSSs that resolve without intervention, and others will develop persistent PTSSs.^14,15^ Findings in related studies indicate that an individual’s PTSS trajectory affects other health outcomes^16,17,18,19^ and subsequent treatment needs.^17,20,21,22^ Emerging evidence suggests that PTSS trajectory can be predicted by baseline characteristics identifiable near the time of traumatic event, such as age, education level, heavy alcohol use, and prior interpersonal injury.^14,23,24^ To our knowledge, PTSS trajectories, which can inform early, personalized interventions for individuals at highest risk for PTSS, have not been previously measured in ICU caregivers except in our prior pilot study.^25^

The primary objective of this investigation was to define 6-month PTSS trajectories for caregivers of adult patients with acute cardiorespiratory failure. We hypothesized that PTSS trajectories would be identified in multiple distinct groups, including improving, worsening, and persistently elevated over time (ie, chronic). Secondary objectives were to (1) identify patient and caregiver characteristics that are associated with caregiver PTSS trajectory group membership and (2) to measure the association between caregiver trajectory group membership and caregiver and patient health outcomes.

## Methods

### Study Design and Participants

We performed a prospective cohort study of patients with acute cardiorespiratory failure admitted to the medical ICU at the University of North Carolina Medical Center, an academic 903-bed hospital located in Chapel Hill, North Carolina. Patients were enrolled in a dyad along with their primary caregiver. Patients were eligible if they required (1) mechanical ventilation via endotracheal tube, (2) high-flow nasal cannula, (3) noninvasive positive pressure ventilation, or (4) hypotension requiring vasopressors or inotropes. Patients both with and without COVID-19 were enrolled. The primary caregiver was defined as the unpaid individual who provided the most physical, emotional, or financial support prior to ICU admission; both legally defined and chosen family relationships were included, and neither cohabitation nor legal relationship were required.^26^ Both patients and caregivers were 18 years or older and had English language proficiency. Written or verbal informed consent for participation was obtained from caregivers; patients provided informed consent at the time of enrollment if decision-making capacity was present or later as part of a re-consent protocol if decision-making capacity was regained. The study protocol was approved by the institutional review board at The University of North Carolina at Chapel Hill. This research study followed the Strengthening the Reporting of Observational Studies in Epidemiology (STROBE) reporting guideline.

### Data Collection

Participant assessment occurred at 4 time points: within the first 48 hours of ICU admission, immediately following ICU discharge, and 3 and 6 months after enrollment. Because of COVID-19–related visitor restrictions that were present in complete or partial form for most of the enrollment period, participants were enrolled remotely with baseline assessment completed either through phone or email. For participants enrolled after restrictions ended, there was an option to complete the initial assessment in person, with 10 of 95 participants (11%) enrolled in person. Participants were contacted by telephone or email for subsequent assessments. Data collection occurred through a combination of caregiver interview, patient interview (if able to participate), and medical record abstraction.

### Outcomes

The primary outcome of the study was caregiver PTSSs, measured using the Impact of Events Scale–Revised (IES-R). The IES-R is a validated instrument that is used to screen for symptoms of posttraumatic stress. The IES-R been used (in original form) to evaluate the experience of families of ICU survivors and nonsurvivors.^11,27,28^ The IES-R contains 22 questions assessing how much the respondent has been bothered by specific PTSSs (such as intrusive thoughts of the traumatic event) over the last 7 days. Each item is scored from 0 (not at all) to 4 (extremely). The total score ranges from 0 to 88, with a higher score indicating more severe PTSSs. A score of 33 or higher indicates symptoms consistent with a probable diagnosis of posttraumatic stress disorder (PTSD).^27,29^ The baseline and 6-month assessments also included the measures described in the following sections.

#### Baseline Assessment

This included patient and caregiver sociodemographic characteristics and chronic comorbidities, patient acute comorbidities, characteristics of the patient-caregiver relationship, caregiver resilience (Connor-Davidson Resilience Scale),^30^ caregiver employment history,^31^ patient prehospital functional status (Functional Independence Measure [FIM]),^32^ caregiver anxiety and depression symptoms (Hospital Anxiety and Depression Scale),^33^ and prior caregiver trauma (Mini International Neuropsychiatric Interview [MINI] PTSD module).^34^

#### Six-Month Assessment

This included assessment of changes to the caregiver’s employment status,^31^ perceived positive aspects of caregiving,^35^ caregiver physical and mental health-related quality of life (36-item Short Form Survey [SF-36], version 2),^36^ and surrogate-determined patient health care utilization, vital status, and functional status (FIM).

### Statistical Analysis

Patient and caregiver characteristics were analyzed using mean and SD for continuous variables and frequency and rate for categorical variables. To identify PTSS trajectories, we performed latent class growth analysis (LCGA).^37^ Our LCGA modeling began with a single-class model with 3 unconditional growth trajectories of PTSS IES-R scores without covariates: one with a constant (flat) trajectory, one with a linear trajectory, and one with a quadratic trajectory. These trajectories were compared based on bayesian information criteria (BIC), which penalizes more complex models when they fail to improve the model fit. The single-class model with the lowest BIC of the 3 trajectories was selected. Next, we looked at 2- to 5-class unconditional LCGA models with the selected trajectory. The best model with the lowest BIC was identified and was extended to examine associations between patient and caregiver characteristics and caregiver trajectory membership in a conditional model. We added 7 covariates for potential association with trajectory group membership chosen a priori based on prior literature and clinical judgment (caregiver age, gender, resilience, prior history of trauma, patient severity of illness, prehospital functional status, and whether visitor restrictions were in place at the time of enrollment) to the 3-class model. When selecting these variables, we considered caregiver race given the established association between belonging to a racialized minority group and increased rates of PTSD.^38,39,40^ However, when considering the mechanism for this association, it seemed more likely that the effects of systemic racism and resulting heightened exposure to lifetime traumatic events were responsible for this association rather than a differing biologic or genetic effect of race.^41^ Thus, to more fully reflect this mechanism and optimize model parsimony, we included prior trauma (witnessing or experiencing actual or threatened death or serious injury) and omitted race.^42^ Finally, we examined associations between caregiver trajectory membership and 6-month caregiver and patient outcomes using the analysis of variance or χ^2^ tests when appropriate. Data from caregivers with at least 1 completed IES-R were included in the analysis, and missing data were not imputed because we assumed the data were missing completely at random and there was no significant evidence suggesting this assumption was violated.

The statistical tests implemented were all 2-sided, and a *P* < .05 was considered significant. We used SAS version 9.4 (SAS Institute) to conduct the statistical analyses, including the trajectory analysis by proc traj (eAppendix in Supplement 1). More detailed statistical analysis from LCGA can be seen in the eAppendix in Supplement 1.

## Results

**Figure 1.**
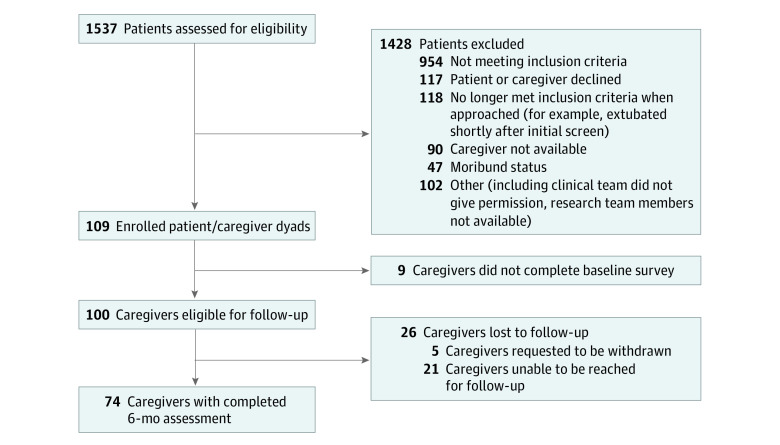
Participant Eligibility and Enrollment

Study participants were screened and enrolled between April 23, 2020, and May 27, 2021. Of the 583 patients who met inclusion criteria, 474 were excluded (Figure 1). A total of 109 patients and their primary caregivers were enrolled over a 13-month period; 9 caregivers did not complete the baseline survey following enrollment and were not contacted for subsequent assessments. Initial PTSS assessment occurred at a mean (SD) of 29.6 (12.6) hours following ICU admission. Five caregivers later requested to be withdrawn. An additional 21 caregivers could not be reached for 1 or more assessments following the baseline survey, leaving 74 of 100 caregivers (74%) with complete 6-month assessments. Caregivers who withdrew, compared with those who did not, reported higher baseline PTSSs (mean [SD] IES-R score, 36.8 [17.4] vs 21.7 [15.7]; *P* < .001).

The mean (SD) age of the 95 enrolled caregivers was 54.2 (13.6) years, 72 (76%) were women, 22 (23%) were Black individuals, and 70 (74%) were White individuals (Table 1). Fifty-six caregivers (59%) responded yes when asked if, prior to the patient’s ICU admission, they had ever experienced, witnessed, or had to deal with a traumatic event, defined as “actual or threatened death or serious injury to you or someone else,” as part of the MINI diagnostic interview. Mean (SD) patient age was 62.3 (16.1) years, and 41 patients (43%) were women (Table 2). The mean (SD) Acute Physiology and Chronic Health Evaluation II score at the time of enrollment was 24.1 (8.1), consistent with a predicted hospital mortality rate of 40%. The mean (SD) ICU length of stay was 9.4 (10.3) days. By 6 months, 40 patients (42%) had died. The mean (SD) FIM score at 6 months for survivors was 111 (26), indicating good functional status.

**Table 1.** Baseline Caregiver Characteristics

Characteristic	Caregivers, No. (%) (N = 95)
Age, mean (SD), y	54.2 (13.6)
Gender	
Women	72 (76)
Men	23 (24)
Race	
Black	22 (23)
White	70 (74)
Other^a^	3 (3)
Hispanic or Latino ethnicity	3 (3)
Employment status	
Working full-time or part-time	57 (62)
Unemployed, on leave, receiving disability, going to school, homemaker	16 (17)
Retired	19 (21)
Education	
High school or less	19 (20)
Associate degree or some college	37 (39)
College or advanced degree	39 (41)
Relationship to patient	
Spouse or partner	36 (38)
Child	33 (35)
Sibling	13 (14)
Parent	9 (9)
Other relative	4 (4)
Religion	
Catholic	7 (7)
Protestant	55 (58)
Jewish	1 (1)
Muslim	2 (2)
None	18 (19)
Other	12 (13)
Caregiving length, y	
<1	41 (43)
1-3	12 (13)
3-5	7 (7)
>5	32 (34)
Do not know or would prefer not to answer	3 (3)
Caregiver living with patient prior to hospital stay	53 (56)
Baseline resilience, mean (SD)^b^	83.4 (11.4)
Baseline Hospital Anxiety and Depression Scale score, mean (SD)^c^	
Total	15.7 (9.6)
Anxiety	9.6 (5.8)
Depression	6.1 (4.7)
Baseline Impact of Events Scale–Revised, mean (SD)^d^	25.0 (17.2)
Previous self-reported mental health diagnosis	
Depression	33 (35)
Anxiety	23 (24)
PTSD	6 (6)
Has ever experienced a traumatic event^e^	56 (59)
Unable to visit loved one on admission due to visitor restrictions	53 (56)

Abbreviation: PTSD, posttraumatic stress disorder.

^a^ Other race included American Indian or Alaska Native (1 individual), Asian (1 individual), and unavailable (1 individual).

^b^ Resilience was measured with the Connor-Davidson Resilience Scale (range, 0-100), with higher scores indicating higher resilience.

^c^ Scores for each subscale range from 0 to 21, with higher scores indicating worse symptoms.

^d^ Scores range from 0 to 88, with higher scores indicating worse symptoms.

^e^ Asked as part of the Mini International Neuropsychiatric Interview PTSD module: “Have you ever experienced or witnessed or had to deal with an extremely traumatic event that included actual or threatened death or serious injury to you or someone else?”

**Table 2.** Baseline Patient Characteristics

Characteristic	Patients, No. (%) (N = 95)
Age, mean (SD), y	62.3 (16.1)
Gender	
Women	41 (43)
Men	54 (57)
Race	
Black	23 (24)
White	67 (71)
Other^a^	5 (5)
Hispanic ethnicity	1 (1)
APACHE II score, mean (SD)	24.1 (8.1)
Chronic comorbidities	
Cancer	22 (23)
Dementia	9 (9)
Chronic liver disease	13 (14)
End-stage kidney disease	7 (7)
Chronic lung disease	23 (24)
Full code at hospital admission	83 (87)
Richmond Agitation Sedation Scale score	
0 (Alert and calm)	44 (46)
+1 to +4 (Restless, agitated, or combative)	11 (12)
−1 to −3 (Drowsy, moderate or light sedation)	30 (32)
−4 to −5 (Deep sedation or unarousable)	10 (11)
CAM-ICU positive^b^	34 (37)
Baseline functional status, mean (SD)^c^	109.7 (28.0)
Admitted with COVID-19	38 (40)
Requiring invasive mechanical ventilation at enrollment	55 (58)

Abbreviations: APACHE II, Acute Physiology and Chronic Health Evaluation II; CAM-ICU, Confusion Assessment Method for the Intensive Care Unit.

^a^ Other race included American Indian or Alaska Native (2 individuals), Asian (2 individuals), and other (1 individual).

^b^ Positive result indicates the presence of delirium.

^c^ Measured with the Functional Independence Measure (range, 0-126), with higher scores indicating better functional status.

### LCGA Results

**Figure 2.**
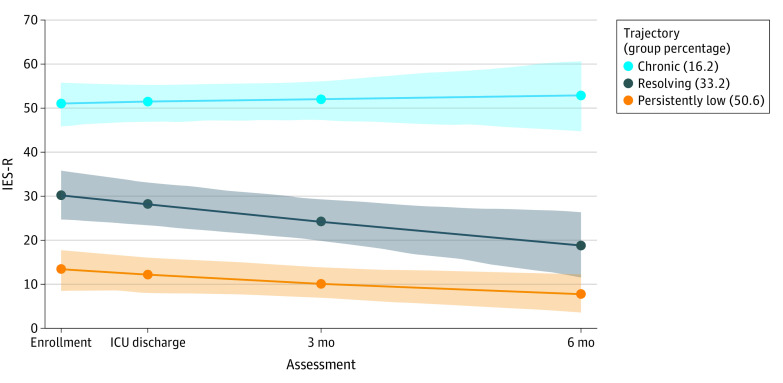
Caregiver Posttraumatic Stress Symptom Trajectories IES-R indicates Impact of Events Scale–Revised; ICU, intensive care unit.

In the first step of selecting the form of the trajectory, we concluded that a linear function had the best fit for the 1-class unconditional LCGA model. Comparing the best single 2- to 5-class models with linear growth curves, we concluded the 3-class model had the best fit with the lowest BIC (2669.8). Accordingly, we identified 3 distinct PTSS trajectories: persistently low, resolving, and chronic (Figure 2). Approximately 16% of caregivers (15) belonged to the chronic trajectory, which was characterized by high PTSSs at initial assessment that remained elevated over time. About one-third of caregivers (31% [29 caregivers]) belonged to the resolving trajectory, characterized by elevated symptoms at baseline that resolved by 6 months. Half of the caregivers (54% [51 caregivers]) did not demonstrate elevated PTSSs at any point.

#### Variables Associated With Trajectory Group Membership

Using backward variable selection, we identified the combination of low baseline caregiver resilience, prior caregiver history of trauma, higher patient severity of illness, and higher baseline patient functional status as having the strongest association with trajectory class membership based on BIC (eAppendix in Supplement 1).

#### Association Between Caregiver PTSS Trajectory and 6-Month Caregiver and Patient Outcomes

Caregivers in the chronic trajectory, compared with caregivers in the persistently low trajectory, reported a lower health-related quality of life at 6 -months (mean [SD] total SF-36 score, persistently low trajectory: 104.7 [11.3]; resolving trajectory: 101.7 [10.4]; chronic trajectory: 84.0 [14.4]; *P* < .001) (Table 3). Caregivers in the chronic trajectory were also more likely to have changes in employment and lower self-perceived effectiveness at work (mean [SD] perceived effectiveness at work, persistently low trajectory: 86.0 [24.2]; resolving trajectory: 59.1 [32.7]; chronic trajectory: 72.3 [18.4]; *P* = .009) after the patient’s ICU admission. We did not identify a statistically significant association between chronic caregiver trajectory and patient mortality (Table 3).

**Table 3.** Six-Month Caregiver and Patient Outcomes by Caregiver Posttraumatic Stress Symptom Trajectory

Characteristic	Mean (SD) score by group			*P* value
	Persistently low (n = 51)	Resolving (n = 29)	Chronic (n = 15)	
Caregiver outcomes				
SF-36^a^				
Total	104.7 (11.3)	101.7 (10.4)	84.0 (14.4)	<.001
Physical Component Score	51.4 (10.0)	50.8 (7.9)	48.5 (13.0)	.82
Mental Component Score	53.4 (7.8)	50.9 (5.9)	35.6 (8.0)	<.001
SF-36 measures of physical health, mean				
Physical functioning	82.2 (23.4)	80.2 (24.2)	65 (38.7)	.17
Role functioning due to physical health	86.3 (23.6)	80.7 (22.4)	66.9 (30.2)	.08
Bodily pain	80.1 (23.4)	68.4 (24.7)	62 (30.2)	.05
General health	69.7 (18.5)	70.1 (22.4)	59.9 (23.1)	.36
SF-36 measures of mental health				
Vitality or energy level	62.0 (20.7)	53.6 (17.6)	23.1 (14.7)	<.001
Social functioning	91.0 (16.4)	88.1 (17.9)	58.8 (32.3)	.001
Role functioning due to emotional health	91.7 (16.4)	82.5 (19.7)	56.7 (25.7)	<.001
Mental health	81.2 (14.5)	75.0 (14.4)	52.5 (16.4)	<.001
Working full- or part-time prior to loved one’s ICU admission, No. (%)	30 (61)	18 (64)	9 (60)	.95
Unable to work at all since patient ICU admission, No. (%)	18 (43)	5 (24)	2 (20)	.19
Perceived effectiveness at work, mean score^b^	86.0 (24.2)	59.1 (32.7)	72.3 (18.4)	.009
Ever had to change jobs because of loved one’s ICU admission, No. (%)	2 (7)	0	4 (44)	.001
Kept same job but had to change work duties because of loved one’s ICU admission, No. (%)	12 (43)	8 (47)	4 (44)	.96
Perceived positive aspects of caregiving, mean score^c^	36.5 (9.2)	33.8 (7.8)	33.5 (9.0)	.45
Patient outcomes, No. (%)				
FIM^d^	114.1 (19.0)	107.1 (36.4)	101.3 (42.5)	.57
Required hospital readmission over the subsequent 6-mo after ICU discharge	10 (30)	4 (25)	0 (0)	.35
Hospital mortality	12 (23)	9 (31)	7 (47)	.22
6-mo mortality	18 (35)	13 (45)	9 (60)	.22

Abbreviations: FIM, Functional Independence Measure; ICU, intensive care unit; SF-36, 36-item Short Form Survey.

^a^ SF-36 assesses 8 distinct health concepts: 4 related to physical function (physical functioning, bodily pain, role limitations due to physical health problems, and general health perceptions) and 4 related to mental health (role limitations due to personal or emotional problems, emotional well-being, social functioning, and energy/fatigue). These 8 subscales are each scored from 0 to 100, with higher scores indicating better health. The subscales are then converted into summary scores for physical health (Physical Component Score) and mental health (Mental Component Score) and normalized so that mean is 50 with an SD of 10. Higher scores indicate better health.

^b^ Effectiveness at work ranges from 0 to 100, with higher scores indicating better self-perceived effectiveness at work.

^c^ Measured with the Positive Aspects of Caregiving Scale (range, 9-45), with higher scores indicating more positive perceptions of caregiving.

^d^ Total score ranges from 0 to 126, with higher scores indicating better functional status.

## Discussion

In this investigation of 6-month PTSS trajectories for caregivers of patients with acute cardiorespiratory failure, we identified 3 distinct patterns of PTSSs over time: persistently low, resolving, and chronic. Sixteen percent of caregivers belonged to the chronic trajectory, and risk factors included low baseline caregiver resilience, caregiver history of trauma, higher patient severity of illness, and higher baseline patient functional status. The chronic trajectory of caregiver PTSSs was associated with worse caregiver outcomes.

To our knowledge, other than our previously published pilot study,^25^ this is the first study to measure PTSS trajectories in medical ICU caregivers. Prior investigations have performed serial assessments of PTSS scores and have found that mean symptom severity decreases over time when a cohort is examined as a whole.^43,44^ However, by using LCGA we identified a subgroup of caregivers with chronic, clinically significant PTSSs and who experienced worse 6-month outcomes. Identifying these caregivers and distinguishing them from caregivers whose PTSS symptom trajectory is less intense or persistent is an essential first step to develop interventions tailored for those caregivers with greatest need of support.

While prior clinical trials of interventions to reduce PTSS among ICU caregivers have enrolled caregivers based on patient characteristics such as severity of illness, our findings suggest that a combination of patient and caregiver characteristics contribute to chronic PTSSs. Our results also indicate potential mechanisms underlying caregiver PTSSs. Prior investigations have identified preexisting caregiver mental health disorder as a risk factor for caregiver PTSSs.^45^ We found a specific association between a caregiver-reported history of trauma and chronic PTSSs; thus, retraumatization in the ICU may be driving the development of PTSSs for some caregivers. Retraumatization has been described as the reaction to a traumatic experience that is intensified or otherwise shaped by a reaction to a previous traumatic experience^46^ and is a risk factor for PTSSs in noncaregiving populations.^47^ Retraumatization and worsening of PTSSs could also explain why some prior caregiver interventions were associated with increased PTSSs.^11,48^ Low resilience has also been identified as a PTSD risk factor in noncaregiving populations,^49,50^ and prior work in caregivers of neuro-ICU patients indicates that low caregiver resiliency might inhibit psychological recovery and promote persistent PTSS.^51^ Notably, we did not find that family visitor restrictions were associated with chronic PTSS. There are several possible explanations for this. Recent literature suggests that long-term outcomes for patients following an ICU stay for COVID-19 are not significantly different from those who are admitted to the ICU for other reasons,^52^ and this may also be the case for their family caregivers. Another possible explanation is that family visitor restrictions are in fact a trigger for caregiver PTSS,^4^ but not one that is more predictive of PTSS trajectory than preexisting characteristics, such as low resilience or a history of trauma. We did not identify a statistically significant association between caregiver PTSS trajectory and patient mortality, which is inconsistent with some prior literature in this area, although it is possible that our analysis did not possess sufficient statistical power to detect such a difference.^5^ Qualitative work with a subset of caregivers enrolled for this study is ongoing to gain more nuanced perspective on the association of factors such as visitor restrictions and patient death with caregiver PTSSs.

Our results have several implications for supporting ICU caregivers. First, caregivers in the chronic trajectory could be detected within the first 48 hours of ICU admission, indicating a role for early screening. This approach is comparable with screening for complicated grief used in palliative care and hospice to allocate bereavement services based on need. The IES-R, used here, is relatively simple and can be administered in less than 10 minutes and could be administered to all caregivers upon ICU admission to identify those at highest risk for significant PTSSs. Second, our finding of an association between low caregiver resilience and chronic PTSSs suggest a role for resiliency-building interventions, such as cognitive behavioral therapy teaching adaptive coping and cognitive flexibility.^53^ Resiliency interventions have been described as potentially efficacious in noncaregiving populations,^54,55^ and a pilot study of a resiliency intervention for caregivers and patients in a neuro-ICU showed promise for reducing caregiver PTSSs.^56^ Finally, our finding that a history of trauma is associated with chronic PTSSs indicates the importance of grounding future caregiver interventions in the principles of trauma-informed care. Trauma-informed care is centered on understanding the impact of trauma in the context of each person’s history and social context and avoiding retraumatization^57^ and is gaining increasing recognition as an essential component of care in the ICU.^58^ Given the promise of the aforementioned therapeutic approaches, incorporation of psychologists into ICU care teams may be warranted.

Our investigation has several strengths. To our knowledge this is the first large study to use LCGA to identify PTSS trajectories in caregivers of medical ICU patients, and our findings fill an important knowledge gap about the heterogeneity of stress and trauma responses in this population.^59^ We enrolled a broader population than was enrolled in many prior investigations of PTSSs in ICU caregivers, allowing for greater generalization to medical ICU patients.

### Limitations

This study has limitations. By enrolling only patients experiencing medical critical illness from a single academic center during the early waves of the COVID-19 pandemic, our findings may not be generalizable to other settings. We had higher study attrition than anticipated, with 74 enrolled caregivers (77%) completing the 6-month assessment. While reasons for this are not clear, the higher study attrition here vs our pilot study suggest that stresses associated with the COVID-19 pandemic resulted in less time or interest in continuing study activities.^25^ Baseline PTSSs were higher among caregivers who withdrew, suggesting a healthy volunteer effect in which the percentage of caregivers belonging to the chronic trajectory may be underestimated.

## Conclusions

In conclusion, we identified 3 distinct PTSS trajectories among ICU caregivers and found that 16% of caregivers experienced chronic PTSSs over the 6 months following a patient’s ICU stay. These individuals had diminished quality of life and reduced effectiveness at work. The chronic trajectory was identified by a combination of low baseline caregiver resilience, prior caregiver history of trauma, higher patient severity of illness, and higher baseline patient functional status. Future interventions for ICU caregivers should include early screening for PTSSs and incorporate therapeutic components tailored toward caregivers with low resilience and a history of trauma.

## Notes

Supplement 1. **eAppendix.** Supplemental Methods
eReferences.

Supplement 2.
Data Sharing Statement
