# Supplementary material for: Posttraumatic Stress Symptom Trajectories in Family Caregivers of Patients With Acute Cardiorespiratory Failure
Source: JAMA Netw Open. 2023 Apr 7;6(4):e237448. doi: 10.1001/jamanetworkopen.2023.7448 (PMC10082401; doi:10.1001/jamanetworkopen.2023.7448)
Supplement: Supplement 1. — eAppendix. Supplemental Methods eReferences. [file jamanetwopen-e237448-s001.pdf]

Supplemental Online Content

Wendlandt B, Pongracz L, Lin F, et al. Posttraumatic stress symptom trajectories in family caregivers of patients with acute cardiorespiratory failure. *JAMA Netw Open.* 2023;6(4):e237448. doi:10.1001/jamanetworkopen.2023.7448

eAppendix. Supplemental Methods

eReferences.

This supplemental material has been provided by the authors to give readers additional information about their work.

© 2023 Wendlandt B et al. *JAMA Network Open.*

eAppendix. Supplementary Methods

The trajectory analy[1]sis in the manuscript is based on the SAS procedure proc traj[2]. We referred the statistical theory behind the procedure to Jones et al[1]. In general, the procedure can find latent classes of members with different trajectories that can be either constant, linear, or quadratic based on a finite-mixture model. Table S1 shows the result of the basic model after model selections using Bayesian Information Criteria (BIC). With the smallest BIC, we determined the optimal linear trajectories for the three-class model. As one can see from the table, Group 1 has the lowest baseline mean values (intercept=12.2) have a linearly down-trended trajectory (linear estimate=-1.13, p-value=0.014). Group 2 has a similar trend (linear estimate=-1.93, p-value=0.02) but has a higher baseline mean value (intercept=30.3) than Group 1. Group 3 has the worse baseline mean value (intercept=50.9) and remains high scores chronically over the follow-up.

Table S1. Parameter estimates, standard errors, and p-values of the basic trajectory model

Group	Parameter	Estimate	Standard Error	p-value
1 (persistently low)	Intercept	12.2	1.74	<0.001
	Linear	-1.13	0.46	0.014
2 (resolving)	Intercept	30.3	2.61	<0.001
	Linear	-1.93	0.63	0.002
3 (persistently high)	Intercept	50.9	2.49	<0.001
	Linear	0.31	0.84	0.711

The procedure can also utilize baseline predictors to predict the membership, i.e., associating the predictors to membership probability in a multinomial logistic regression model. As the manuscript indicated, we started with seven predictors and used BIC to select variables. Table S2 shows that the model with predictors of patient severity of illness, baseline caregiver resilience, baseline patient functional status, and prior caregiver history of trauma has the smallest BIC when adding them to the three-class basic model in Table S1. The result shows that an individual with a higher baseline patient functional status is more likely to be in Group 2 (resolving) than Group 1 (persistently low)(estimate=0.113, p-value=0.020), an individual with less baseline caregiver resilience is more likely to be in Group 3 (persistently high) than Group 1 (persistently low) (estimate=-0.074, p-value=0.024), and an individual with prior caregiver history of trauma is more likely to be in Group 3 (persistently high) than Group 1 (persistently low) (estimate=1.985, p-value=0.035).

Table S2. Parameter estimates, standard errors, and p-values of the basic trajectory model

Group	Parameter	Estimate	Standard Error	p-value
1 (persistently low)	Intercept	13.1	1.55	<0.001
	Linear	-1.22	0.46	0.008
2 (resolving)	Intercept	32.8	2.57	<0.001
	Linear	-2.37	0.69	0.001
3 (persistently high)	Intercept	50.9	2.61	<0.001
	Linear	0.43	0.84	0.608
Group membership				
2 vs. 1	Constant	-11.69	6.207	0.061

© 2023 Wendlandt B et al. *JAMA Network Open.*

	patient severity of illness	0.035	0.038	0.362
	baseline caregiver resilience	-0.043	0.031	0.168
	baseline patient functional status	0.113	0.048	0.020
	prior caregiver history of trauma	0.521	0.700	0.457
3 vs. 1	Constant	0.186	2.620	0.943
	patient severity of illness	0.069	0.044	0.117
	baseline caregiver resilience	-0.074	0.033	0.024
	baseline patient functional status	0.015	0.014	0.280
	prior caregiver history of trauma	1.985	0.936	0.035

Sample size: We targeted a sample of 100 patients and their primary caregivers. Based on previous experience in our research group, we estimated it was feasible to enroll 100 caregivers over 12 months[3]. A targeted sample size of 100 caregivers with 10-15% anticipated dropout provides a cohort of approximately 90 caregivers. While it was not known how many classes would be identified or how many caregivers would belong to each class, with a sample of 85 caregivers in four classes, with 24 caregivers in each class, we anticipated detecting a medium effect size of 0.35 in Cohen's f between the four classes for a normally distributed outcome under 0.8 power and 0.05 type-I error rate. If two classes werefound with 45 caregivers in each class, we anticipated detecting a medium effect size of 0.3 in Cohen's f between the two classes.

© 2023 Wendlandt B et al. *JAMA Network Open.*

eReferences

1. Jones B, Nagin, D., & Roeder, K. A SAS Procedure Based on Mixture Models for Estimating Developmental Trajectories. Sociological Methods & Research (2001) 29: 374-393.
2. SAS® Proc Traj Home. http://www.andrew.cmu.edu/user/bjones.
3. Wendlandt B, Ceppe A, Gaynes BN, et al. PostWendlandt B, Chen YT, Lin F-C, et al. Posttraumatic Stress Disorder Symptom Trajectories in ICU Family Caregivers. *Critical care explorations*. 2021;3(4):e0409-e0409. doi:10.1097/CCE.0000000000000409

© 2023 Wendlandt B et al. *JAMA Network Open.*
