# Supplementary material for: Posttraumatic Stress Symptom Trajectories in Family Caregivers of Patients With Acute Cardiorespiratory Failure
Source: JAMA Netw Open. 2023 Apr 7;6(4):e237448. doi: 10.1001/jamanetworkopen.2023.7448 (PMC10082401; doi:10.1001/jamanetworkopen.2023.7448)
Supplement: Supplement 2. — Data Sharing Statement [file jamanetwopen-e237448-s002.pdf]

Data Sharing Statement

Wendlandt. Posttraumatic Stress Symptom Trajectories in Family Caregivers of Patients With Acute Cardiorespiratory Failure. *JAMA Netw Open*. Published April 07, 2023. doi:10.1001/jamanetworkopen.2023.7448

Data
Data available: Yes
Data types: Deidentified participant data, Data dictionary
How to access data: blair.wendlandt@unchealth.unc.edu
When available: With publication

Supporting Documents
Document types: Statistical/analytic code, Informed consent form
How to access documents: blair.wendlandt@unchealth.unc.edu
When available: With publication

Additional Information
Who can access the data: Researchers whose proposed use of the data has been approved.
Types of analyses: Each analysis request will be considered and approved on an individual basis.
Mechanisms of data availability: After approval of a proposal and with a signed data access agreement.
Any additional restrictions: Given the sensitive nature of the data collected, the investigators will consider requests for sharing on a case-by-case basis.
